# Treatment of asymptomatic vaginal candidiasis in pregnancy to prevent preterm birth: an open-label pilot randomized controlled trial

**DOI:** 10.1186/1471-2393-11-18

**Published:** 2011-03-11

**Authors:** Christine L Roberts, Kristen Rickard, George Kotsiou, Jonathan M Morris

**Affiliations:** 1 Kolling Institute of Medical Research, University of Sydney, NSW Australia; 2 Department of Obstetrics and Gynaecology, Royal North Shore Hospital, NSW Australia; 3 Department of Microbiology and Infectious Diseases, Royal North Shore Hospital, NSW Australia

## Abstract

**Background:**

Although the connection between ascending infection and preterm birth is undisputed, research focused on finding effective treatments has been disappointing. However evidence that eradication of *Candida *in pregnancy may reduce the risk of preterm birth is emerging. We conducted a pilot study to assess the feasibility of conducting a large randomized controlled trial to determine whether treatment of asymptomatic candidiasis in early pregnancy reduces the incidence of preterm birth.

**Methods:**

We used a prospective, randomized, open-label, blinded-endpoint (PROBE) study design. Pregnant women presenting at <20 weeks gestation with singleton pregnancies self-collected a vaginal swab. Those who were asymptomatic and culture positive for *Candida *were randomized to 6-days of clotrimazole vaginal pessaries (100mg) or usual care (screening result is not revealed, no treatment). The primary outcomes were the rate of asymptomatic vaginal candidiasis, participation and follow-up. The proposed primary trial outcome of spontaneous preterm birth <37 weeks gestation was also assessed.

**Results:**

Of 779 women approached, 500 (64%) participated in candidiasis screening, and 98 (19.6%) had asymptomatic vaginal candidiasis and were randomized to clotrimazole or usual care. Women were not inconvenienced by participation in the study, laboratory testing and medication dispensing were problem-free, and the follow-up rate was 99%. There was a tendency towards a reduction in spontaneous preterm birth among women with asymptomatic candidiasis who were treated with clotrimazole RR = 0.33, 95%CI 0.04-3.03.

**Conclusions:**

A large, adequately powered, randomized trial of clotrimazole to prevent preterm birth in women with asymptomatic candidiasis is both feasible and warranted.

**Trial registration:**

Australia and New Zealand Clinical Trials Register (ANZCTR): ACTRN12609001052224

## Background

Prevention of spontaneous preterm birth remains one of the most important challenges in modern maternity care. Whilst an association between ascending infection and preterm birth is undisputed, research focussed on finding effective preventive treatments has been disappointing[1]. To date, most treatment trials (e.g. for bacterial vaginosis, *Ureaplasma urealyticum*, *Chlamydia trachomatis*, trichomoniasis) have found little effect on the rate of preterm birth[2]. In contrast, in a randomized controlled trial of early antenatal screening (15-19 weeks) and treatment for asymptomatic bacterial vaginosis, candidiasis and/or trichomoniasis in early pregnancy, Kiss and colleagues reported a 46% reduction in the spontaneous preterm birth rate[3]. Post-hoc subgroup analyses of this trial suggest the benefit was primarily among those women who were treated for asymptomatic candidiasis.

The results of observational studies of the association between candidiasis and preterm birth have been mixed. Two cohort studies conducted in high-risk obstetric populations in the United States in the 1980 s found no significant association between preterm birth and moderate to heavy growth of *Candida albicans *or other *Candida *species at 22-30 weeks gestation[4,5], In contrast, retrospective analyses of the prevalence of preterm birth in population-based data from Hungary found that vaginal clotrimazole treatment of candidiasis during pregnancy was associated with a significantly higher mean gestational age, resulting in a 34-64% reduction in the prevalence of preterm birth[6-8]. A similar reduction in preterm birth (49%) was observed in retrospective study of Latina women in New York who were treated with intravaginal azoles for *Candida *vaginitis[9]

Pregnant women have a two-fold increase in the prevalence of vaginal colonization by *Candida *species compared with non-pregnant women[10]. This association is influenced by increased levels of circulating oestrogens and deposition of glycogen and other substrates in the vagina during pregnancy[10]. Trials of treatment of candidiasis in pregnancy have been limited to women with symptomatic candidiasis (thrush) and the outcome limited to successful eradication of *Candida *colonization and amelioration of symptoms, not pregnancy outcomes[11]. Women with symptomatic candidiasis cannot ethically be randomized to a 'no treatment' trial arm, whereas currently, asymptomatic *Candida *colonization is not considered to require treatment. Consequently, we undertook a pilot study to determine the feasibility of conducting a large randomized controlled trial to answer the clinical question 'In pregnant women with asymptomatic candidiasis does treatment with clotrimazole reduce the incidence of preterm birth?' In addition to assessing procedural feasibility, the aim of the pilot study was to determine the rate of asymptomatic vaginal candidiasis <20 weeks gestation and at 24-28 weeks gestation in an unselected maternity population.

## Methods

This open-label pilot study was conducted in a single Australian tertiary obstetric hospital with recruitment between May 2008 and December 2009. Eligible women included those with a singleton pregnancy of 12 to 19 weeks gestation, aged 18 years or over, no symptoms of vaginal candidiasis at the time of recruitment and no known sensitivity to clotrimazole. Women were recruited by research midwives during a routine antenatal clinic visit. All women provided written informed consent prior to entry into the study. Participants were advised at study entry to seek medical attention from their local doctor or other antenatal care provider if symptoms of thrush developed at any stage in their pregnancy.

Baseline data (age, gravidity, parity and previous preterm birth) were collected at the time of obtaining informed consent and women were instructed on the self-collection of a vaginal swab for isolation of *Candida *species. Such self-collection is commonly used for screening in pregnancy[12]. All women with *Candida *isolated on the initial swab and a random sample of women without candidiasis were asked to provide a second vaginal swab at 24-28 weeks.

All vaginal swabs were received within 24 hours of collection and inoculated onto full plates of chromogenic agar (CHROMagar™Candida, CHROMagar, Paris, France) to isolate, provisionally speciate and semi-quantitatively enumerate *Candida *species (scant, moderate, or heavy). Cultures were incubated for 72 hours at 35 degrees Celsius in 5% CO_2_. Identification to species level was based on colony morphology and color.

### Pilot randomized controlled trial

Women with asymptomatic *Candida *colonization were randomized (central telephone randomisation) to receive either clotrimazole (Canestan^®^) 100 mg vaginal pessaries for 6 nights) or usual care (screening result not revealed, no treatment, routine antenatal care). The clotrimazole could be collected from the hospital, or where this was not feasible it was mailed.

This was a prospective, randomized, open-label, blinded-endpoint (PROBE) trial[13]. Although women and their care providers were not informed of swab results, those randomized to the treatment group were aware of the result because of their allocation to receive medication. The primary trial outcome was birth <37 weeks gestation following spontaneous onset of labor or following preterm prelabor rupture of membranes (PPROM). Gestational age was based on a 1^st ^trimester ultrasound scan. Other outcomes (pregnancy complications, elective preterm delivery, mode of delivery and infant outcomes) were abstracted from the medical records.

### Sample size and statistical analysis

A sample size of 500 women was selected to determine the rate of asymptomatic candidiasis, pilot test the study procedures, and help inform the recruitment numbers and sample size for a definitive trial. The overall colonization rate and its 95% confidence interval (CI) were determined and descriptive analyses of women with and without asymptomatic candidiasis were undertaken. Differences between the two groups were assessed by chi-square tests with the P value set at 0.05.

All trial analyses were by intention to treat, including one woman without candidiasis who was inadvertently randomized to the usual care group. The relative risk (RR) and 95% confidence interval (95%CI) of preterm birth and other outcomes were calculated for women assigned to clotrimazole compared to usual care. The study was approved by the Northern Sydney Central Coast Health Service Ethics Committee.

## Results

### Screening for asymptomatic candidiasis

Of 779 women approached, 500 (64%) agreed to participate in the study. For one woman, the swab result was not obtained. Of the remaining 499 women, 98 (19.6%, 95%CI 16.2-23.1%) women had asymptomatic candidiasis. Women with asymptomatic candidiasis were similar to those women without candidiasis with respect to age, gravidity, parity and history of previous preterm birth (Table 1). The overall preterm birth rate among those women *without Candida *colonization was 3.7%, including 3.0% with spontaneous preterm birth or PPROM. Of 28 women *without *candidiasis at enrolment who had a 2^nd ^vaginal swab, one had scant *Candida albicans *at 24-28 weeks.

**Table 1:** Characteristics of asymptomatic women by *Candida *colonization

Maternal baseline characteristics	Colonized N = 98 n (%)	Not colonized N = 400 n (%)	P-value
Age (years)			
18-34	64 (65)	234 (59)	0.22
≥ 35	34 (35)	166 (41)	
Gravidity/parity			
Nulligravida	25 (26)	125 (31)	0.27
Nullipara	19 (18)	53 (13)	
Multipara	54 (55)	222 (56)	
Previous preterm birth	4 (4)	17 (4)	0.94

Gravidity = number of pregnancies

Parity = number of births ≥20 weeks or ≥400 grams

Of the women with candidiasis, 72 (73%) were colonized with *Candida albicans*, 14 (14.3%) *Candida glabrata*, 5 (5.1%) *Candida parapsilosis*, 3 (3.1%) each of *Candida krusei *and *Candida tropicalis *and 1 (1%) *Trichosporon inkin *species. Semi-quantitative enumeration was reported for 94 specimens; 48 (51%) scant, 34 (36%) moderate and 12 (13%) heavy colonisation.

### Pilot randomized controlled trial

Fifty women with asymptomatic candidiasis were randomized to clotrimazole and 49 to usual care. The baseline characteristics of each group varied consistent with the small number randomized (Table 2). One woman in the usual care group moved interstate and was lost to follow-up. Trial outcomes are reported in Table 3. Five women delivered preterm, 2 (4.0%) in the clotrimazole group and 3 (6.3%) in the usual care group. Except for one woman in the clotrimazole group who was delivered electively for preeclampsia, all followed PPROM and/or spontaneous preterm labour (RR = 0.33 95%CI 0.04-3.03). There were no infants with an Apgar score <7 at 5 minutes and no perinatal deaths.

**Table 2:** Baseline characteristics of women randomized to clotrimazole and usual care

Maternal baseline characteristics	Clotrimazole N = 50 n (%)	Usual care N = 49 n (%)
Age (years)		
18-34	28 (56)	36 (73)
≥ 35	22 (44)	13 (27)
Gravidity/parity		
Nulligravida	11 (22)	14 (29)
Nullipara	9 (18)	11 (22)
Multipara	30 (60)	24 (49)
Previous preterm birth	2 (4)	2 (4)
Candidiasis*		
Candida albicans	37 (74)	35 (73)
Moderate or heavy growth	27 (55)	19 (42)

Gravidity = number of pregnancies

Parity = number of births ≥20 weeks or ≥400 grams

* Excludes five women (1 clotrimazole and 4 no treatment) for whom growth information was not reported

**Table 3:** Trial outcomes for women randomized to clotrimazole and usual care

Trial outcomes	Clotrimazole N = 50 n (%)	Usual care N = 49* n (%)	RR (95% CI)
Preterm birth			
Spontaneous/PPROM	1 (2)	3 (6)	0.33 (0.04, 3.03)
Any preterm birth	2 (4)	3 (6)	0.65 (0.11, 3.74)
Pregnancy complications			
Gestational diabetes	6 (12)	5 (10)	1.18 (0.38, 3.60)
Antepartum haemorrhage/abruption	2 (4)	4 (8)	0.49 (0.09, 2.55)
Induction of labor Mode of delivery	16 (32)	11 (22)	1.43 (0.74, 2.75)
Spontaneous vaginal	21 (42)	20 (41)	1.03 (0.64, 1.64)
Instrumental	9 (18)	10 (20)	0.88 (0.39, 1.98)
Caesarean section	20 (40)	18 (37)	1.09 (0.66, 1.80)
Birthweight (grams)			
< 2500	2 (4)	2 (4)	0.98 (0.14, 6.68)
2500-3999	42 (84)	42 (86)	0.98 (0.83, 1.16)
≥4000 gms	6 (12)	4 (8)	1.47(0.44, 4.89)
Nursery admission	4 (8)	3 (6)	1.31 (0.31, 5.54)

Relative risk (RR) and 95% confidence interval ((95% CI) of the outcomes in the clotrimazole versus usual care group

PPROM - preterm prelabor rupture of the membranes

* One woman who was lost to follow-up is included in the denominator

Ninety five (96%) women with asymptomatic candidiasis had a 2^nd ^vaginal swab at 24-28 weeks. The mean duration between first and second vaginal swabs was 10 weeks in both arms of the trial. The colonization rates at 24-28 weeks were: 49% in those treated with clotrimazole, with the rate of moderate-heavy colonization 55% at enrolment and 42% at 24-28 weeks (P = 0.3); 76% in those in the usual care group with moderate-heavy colonization rates of 42% and 40% respectively. For women with candidiasis both at enrolment and at 24-28 weeks the *Candida *species remained the same except for one woman in the no treatment group where the species changed from *C. glabrata *to *C. kefyr*.

## Discussion

We have demonstrated the feasibility of performing a randomized controlled trial of clotrimazole for pregnant women with asymptomatic candidiasis. The participation rate (64%) was acceptable, women were not inconvenienced by participation in the study, laboratory testing and medication dispensing were problem free, and the follow-up rate was 99%. Although there were no statistically significant differences in outcomes between treatment groups, the findings were in the direction of a treatment effect.

We chose a PROBE design to overcome some specific disadvantages associated with a double-blind, placebo-controlled trial for answering our clinical question[13,14]. First, for our hypothesis, a placebo arm would not represent current clinical practice (no screening and no treatment). Second we were concerned that knowledge of *Candida *colonization might change participants behavior such that they would seek active therapy (clotrimazole vaginal preparations are available over the counter in Australia). And finally, as a vaginally administered placebo will necessarily contain an alcohol preservative, it may be biologically active and have an independent effect on vaginal flora.

Findings from this pilot study support the hypothesized role of *Candida *in the causal pathway to preterm birth. First, there was a higher spontaneous preterm birth rate in women with untreated asymptomatic candidiasis compared to those without candidiasis (6.25% versus 2.99%, RR = 2.2 95%CI 0.5-8.7) consistent with *Candida *colonization as a risk factor for preterm birth[3,15,16]. Second, there was a tendency towards a reduction in preterm birth for those women treated with clotrimazole, consistent with that reported by Kiss[3], Although the randomized trial design minimises bias by balancing both known and unknown prognostic factors in the assignment of treatments[17], the wide confidence intervals around the estimates are also consistent with a null finding or one of increased risk.

This pilot study established the rate of asymptomatic vaginal colonization with *Candida *early in pregnancy (20%) and at 24-28 weeks (76% untreated and 49% treated, P < 0.01). Our observed colonization rate of 20% is consistent with the few published studies. Kiss reported a 14% colonization rate at 15-19 weeks gestation in asymptomatic women with singleton pregnancies[3]. A multicentre prospective US study of an ethnically diverse population enrolled at 23-26 weeks gestation reported that 10% of women had moderate-heavy colonization with *Candida *and among a subset from a single centre the total *Candida *colonization rate was 22% (58% light and 42% moderate-heavy colonization) with 87% *C albicans *and 94% of women asymptomatic[4]. Two other single centre US studies reported candidiasis rates at 22-30 weeks gestation of 14-38% in diverse populations of pregnant women[5,18].

The post-treatment colonization rate of 48% in our pilot study was higher than most, but not all (median 22%, range 0-73%) treatment trials of symptomatic (culture positive) vaginal candidiasis (thrush)[11]. The latter trials varied widely in gestational age at enrolment (14-36 weeks gestation), dose and type of imidazole (1%-2%, clotrimazole, miconazole & econazole), duration of imidazole therapy (1-14 days) and the duration between treatment and follow-up [11]. Importantly in the pilot study, the 10-week average duration between the enrolment and 24-28 week culture was notably longer than the thrush treatment trials (1-5 weeks)[11]. We cannot determine whether treatment led to eradication followed by recolonization or simply a reduction in the level of *Candida *growth in individual participants. A larger trial could determine whether the observed trend to a reduced level of moderate-heavy *Candida *colonization (55% to 42% light colonisation) among treated women occurred by chance or was a true effect.

We considered carefully the timing of the intervention and chose to screen for and treat candidiasis in the early/mid part of the second trimester for a number of reasons. First, early intervention is consistent with the protocol in the Kiss trial[3] in which screening and subsequent treatment of candidiasis occurred between 15 weeks and 19 weeks gestation. Second, accumulating evidence suggests that the role of infection in preterm birth is a chronic process.^4 ^Organisms detected in the uterus before membrane rupture are typically of low virulence, probably accounting for both the chronicity of intrauterine infections and the frequent absence of overt clinical signs of infection.^4 ^And third, although the trials of treatment of bacterial vaginosis overall demonstrate no benefit in the reduction of preterm birth, a positive treatment effect is seen in those trials that initiated treatment early in the second trimester[2]. The current Cochrane Review on bacterial vaginosis concludes that: "treatment before 20 weeks' gestation may reduce the risk of preterm birth. This needs to be further verified by future trials." If early pregnancy is the period of vulnerability to establishment of inflammatory responses to low virulence organisms that will increase the risk of preterm birth, then candidiasis late in pregnancy may be unimportant.

The safety of any proposed intervention in pregnancy is of paramount importance. Clotrimazole is classified as a Category A drug, which has been used by a large number of pregnant women without any proven increase in the frequency of malformations or other direct or indirect harmful effects on the fetus having been observed[19,20]. Local application of clotrimazole vaginal pessaries or cream is generally well tolerated although skin reactions (burning, stinging, or redness) can occur occasionally. We chose a 6-day treatment (rather than shorter course) because this is supported by the Cochrane Systematic Review of treatment for *Candida *eradication in pregnancy[11] and was the regimen used in the Kiss trial[3].

## Conclusions

We believe a large, adequately powered, randomized trial of clotrimazole to prevent preterm birth in women with asymptomatic candidiasis is both feasible and warranted. This pilot study has informed the development and funding application for such a trial[21]. Should this intervention prove effective, a screening protocol could be readily incorporated into routine antenatal care and treatment is available and widely accepted. If it can be demonstrated that treatment of asymptomatic candidiasis reduces preterm births this will change current practice internationally and will directly impact the management of every pregnant woman.

## Competing interests

The authors declare that they have no competing interests.

## Authors' contributions

JM conceived the project and all the authors contributed to design of the study. GK had oversight of the microbiological aspects of the study. CR and KR initially drafted the manuscript and all authors were involved in critical revision of the intellectual content. All authors approved the final manuscript.

## Pre-publication history

The pre-publication history for this paper can be accessed here:

http://www.biomedcentral.com/1471-2393/11/18/prepub
